# Mucinous cystic neoplasms of the pancreas demonstrate *in situ* production of estrogen

**DOI:** 10.3389/fcell.2025.1684564

**Published:** 2025-11-17

**Authors:** Jinyong Kim, Aswath P. Chandrasekar, Benjamin J. Van Treeck, Maria C. Olave, Maryam Shahi, Roger K. Moreira, Seul Kee Byeon, Gunveen S. Sachdeva, Justin E. Juskewitch, Jamie N. Bakkum-Gamez, Scott H. Kaufmann, Sun-Hee Lee, Michelle D. Reid, Maria Westerhoff, Daniela S. Allende, Akhilesh Pandey, Rondell P. Graham

**Affiliations:** 1 Department of Laboratory Medicine and Pathology, Mayo Clinic, Rochester, MN, United States; 2 Division of Gynecologic Oncology Surgery, Mayo Clinic, Rochester, MN, United States; 3 Division of Oncology Research, Mayo Clinic, Rochester, MN, United States; 4 Department of Pathology, Emory University Hospital, Atlanta, GA, United States; 5 Department of Pathology, University of Michigan, Ann Arbor, MI, United States; 6 Department of Pathology, Cleveland Clinic, Cleveland, OH, United States; 7 Center for Individualized Medicine, Mayo Clinic, Rochester, MN, United States; 8 Manipal Academy of Higher Education (MAHE), Manipal, Karnataka, India; 9 Institute of Bioinformatics, International Technology Park, Bengaluru, Karnataka, India

**Keywords:** pancreatic cancer, estrogen, tumor microenvironment, mass spectrometry, tumor biology

## Abstract

**Introduction:**

Mucinous cystic neoplasms (MCNs) are rare cystic tumors that may occur in the liver, pancreas, or retroperitoneum, defined histologically by the presence of an “ovarian type stroma.” While this morphology has been well characterized, it remains unknown whether the tumor stroma is functionally active. In our current study, we describe the detection of estrogen and its metabolites in the ovarian-type stroma of MCN tumors of the pancreas.

**Methods:**

Using a novel liquid chromatography-tandem mass spectrometry (LC-MS/MS) technique. We performed LC-MS/MS on 14 cases of MCN, with and without dysplasia, following macro dissection from formalin fixed tissue.

**Results:**

We identified that relative to histologically normal pancreas, and intraductal papillary mucinous neoplasm (IPMN), the stroma in MCN expresses significantly greater levels of estrone (E1), estradiol (E2), estriol (E3), 2-hydroxyestradiol (2-OHE2), 2-methoxyestrone (2-MeOE1), 2-methoxyestradiol (2-MeOE2) and 16α-hydroxyestrone (16α-OHE1), at levels similar to those seen in the stroma in the normal ovary.

**Discussion:**

These findings establish the functional capability of the ova rian-type stroma in MCN tumors for endogenous hormone production and show that the levels of estrogen in the stroma of MCN tumors approach those of the ovary. These findings serve as a basis for future studies examining the systemic effects of estrogen and the effects of estrogen on tumor progression, both in MCN tumors and tumor metastatic to the ovary.

## Introduction

Mucinous cystic neoplasms (MCNs) are a group of rare cystic tumors that may occur in the liver, pancreas, or retroperitoneum (Crippa et al., 2008; Van Treeck et al., 2020). These tumors occur almost exclusively in women, and a large proportion of tumors encountered are benign, with surgical excision offering an excellent prognosis (Crippa et al., 2008; Griffi et al., 2017). A subset of resected tumors displays high-grade dysplasia or invasive adenocarcinoma (with estimates ranging between approximately 9% and 20%) (Crippa et al., 2008; Griffi et al., 2017; Yamao et al., 2011), which is correlated with increased mortality. The observation that tumors of higher grade and larger size are more frequently encountered in patients of more advanced age (Crippa et al., 2008) lends support to the theory that these tumors exist on a spectrum and progress from benign to malignant lesions over time (as is suggested in primary mucinous cystadenomas of the ovary (Brown and Frumovitz, 2014)); however, the exact mechanisms underlying this progression are unknown.

The defining histologic characteristic of MCNs is the presence of an ovarian-type stroma (Nilsson et al., 2016). The cellular events that lead to the development of this stroma and the resultant MCN tumors have, thus far, been poorly understood. Several theories have been postulated to explain their origin. It has been suggested that the cysts may form as a result of the proliferation of endodermally derived epithelium and mesenchyme in response to female sex hormone stimulation. Alternatively, it has been suggested that these tumors may be a result of local hormone secretion by ectopic ovarian stroma derived from primordial ovarian cells deposited during embryological development (Foster et al., 2015; Zamboni et al., 1999).

Recent studies on the analogous mucinous cystic neoplasm of the liver have established that this stroma is not only histologically similar to the ovary but also exhibits expression of ovarian and sex cord stromal markers, both at the transcriptional and protein levels (Van Treeck et al., 2020). The expression of steroid hormone receptors and steroidogenic enzymes has also been demonstrated in MCNs of the pancreas (Ishida et al., 2016). It remains unclear, however, whether these signatures lead to the active production of estrogen or its metabolites. This demonstration *in situ* would provide insights into the biological significance of the ectopic ovarian-type stroma. Ovarian stroma in the ovary and in the vicinity of ovarian epithelial neoplasms has previously been demonstrated to have significantly increased the expression of markers of sex-steroid differentiation, steroidogenesis, and be capable of producing endogenous estrogen, contributing variably to tumorigenesis and elevated serum estrogen levels (Tokunaga et al., 2007; Kato et al., 2013; Blanco et al., 2017).

Mass spectrometry coupled with gas or liquid chromatography has been used as a technique for the identification and quantification of endogenous estrogen and estrogen metabolites in biological samples (Gouveia et al., 2013). In particular, liquid chromatography–tandem mass spectrometry (LC-MS/MS) has emerged as a preferred method for the analysis of steroid metabolites because this technique is more universally applicable to diverse biological samples and provides high sensitivity and selectivity (Li and Franke, 2015). To date, LC-MS/MS methods have been developed for estrogen and its metabolites in the context of complex biological samples such as serum (Fuhrman et al., 2014), plasma (Penning et al., 2010), urine (Zhang et al., 2022), and fresh-frozen tissues (Blonder et al., 2008; Taioli et al., 2010). However, although other metabolites have been detected from formalin-fixed and paraffin-embedded (FFPE) tissue (Cacciatore et al., 2017; Neef et al., 2020), there are no reports describing the detection of estrogen and estrogen metabolites from FFPE samples using mass spectrometry. In this study, we developed a targeted LC-MS/MS method for detecting estrogen and estrogen metabolites from pancreatic tissue, capable of detecting estrogen metabolites at low femtomole levels (Li and Franke, 2015; Penning et al., 2010; Taioli et al., 2010).

The main objective of this study was to test the hypothesis that the stroma in MCNs is functionally active and capable of producing estrogen and its metabolites and that a novel mass spectrometric technique could detect estrogen signatures in FFPE tissue samples.

## Materials and methods

### Case selection

**FIGURE 1:**
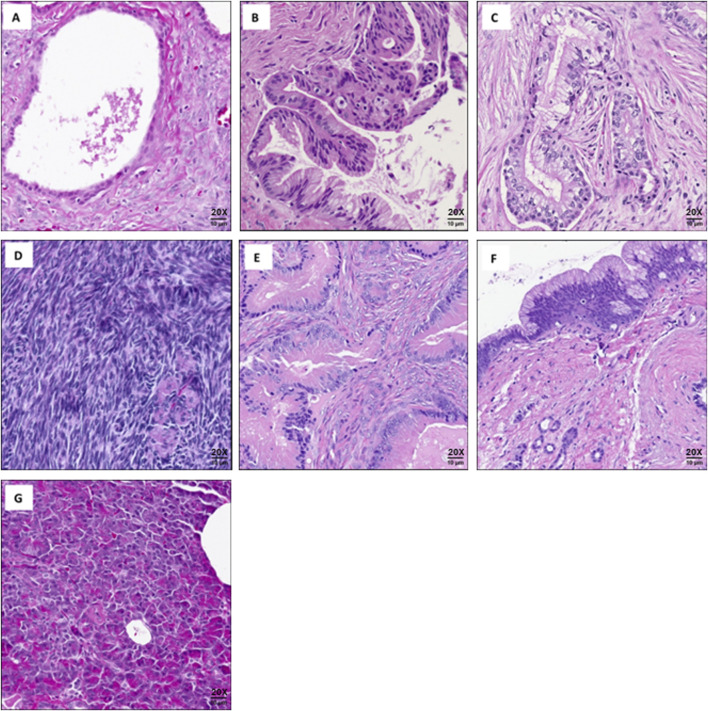
Representative images of cases used for mass spectrometry analysis: **(A)** Low-grade mucinous cystic neoplasm of pancreas. **(B)** High-grade mucinous cystic neoplasm of pancreas. **(C)** Mucinous cystic neoplasm of pancreas with adenocarcinoma. **(D)** Normal ovarian tissue. **(E)** Colorectal cancer metastatic to the ovary. **(F)** Intraductal papillary mucinous neoplasm (IPMN). **(G)** Normal pancreatic tissue. Scale bars are approximate. All images captured at (20X).

We evaluated 14 cases of MCNs of the pancreas, which were selected from our institutional database. The study included two MCNs (2/14) with adenocarcinoma, three with high-grade dysplasia (3/14), and nine (9/14) low-grade MCNs. Five sections of histologically normal pancreas from five patients were used as controls, including two slides (2/5) from patients who had MCNs elsewhere in the pancreas as internal controls. Additionally, to differentiate MCN tumors from other mucinous neoplasms, four cases of intraductal papillary mucinous neoplasm (IPMN) were analyzed. As controls for estrogen detection, ovarian tissue from three premenopausal patients with non-malignant diagnoses and four cases of colorectal cancer (CRC) metastatic to the ovary were also analyzed. Cases were not excluded based on treatment prior to surgery or the use of hormonal therapy (Table 1). Slides from the FFPE tissue were used for the downstream mass spectrometry analyses. Representative images of the analyzed cases are provided in Figure 1.

**TABLE 1:** Clinical characteristics of tissue used for mass spectrometric analysis.

Case number	Age	Sex	Histological diagnosis	Greatest tumor dimension
1	33	F	Low-grade MCN	11.2 cm
2	38	F	Low-grade MCN	11.5 cm
3	58	F	Low-grade MCN	7.9 cm
4	41	F	Low-grade MCN	3.5 cm
5	28	F	Low-grade MCN	1.9 cm
6	46	F	Low-grade MCN	4.3 cm
7	44	F	Low-grade MCN	1.1 cm
8	68	F	Low-grade MCN	3.2 cm
9	47	F	Low-grade MCN	2.4 cm
10	57	F	High-grade MCN	2.0 cm
11	69	F	High-grade MCN	3.7 cm
12	61	F	High-grade MCN	4.8 cm
13	52	F	MCN with invasive carcinoma	5.6 cm
14	34	F	MCN with invasive carcinoma	14.8 cm
15	67	F	IPMN	5.2 cm
16	80	F	IPMN	N/A ^(+)^
17	67	F	IPMN	5.8 cm
18	71	F	IPMN	3.6 cm
19	33	F	Non-neoplastic pancreas *	-
20	50	F	Non-neoplastic pancreas	-
21	68	F	Non-neoplastic pancreas *	-
22	64	F	Non-neoplastic pancreas	-
23	34	F	Non-neoplastic pancreas	-
24	44	F	CRC to ovary	-
25	44	F	CRC to ovary	-
26	59	F	CRC to ovary	-
27	69	F	CRC to ovary	-
28	33	F	Ovary within normal limits	-
29	34	F	Ovary within normal limits	-
30	25	F	Ovary within normal limits	-

Fourteen tissue sections containing mucinous cystic neoplasms, including three cases with high-grade dysplasia and two with adenocarcinoma; three sections of ovarian tissue from premenopausal patients, with non-malignant diagnoses; and five sections of histologically unremarkable pancreatic tissue were used for mass spectrometric analysis. “*”, patient had low-grade MCN elsewhere in the pancreas. (+), multiple masses present.

### Extraction and dansylation of estrogen metabolites from FFPE tissue

**FIGURE 2:**
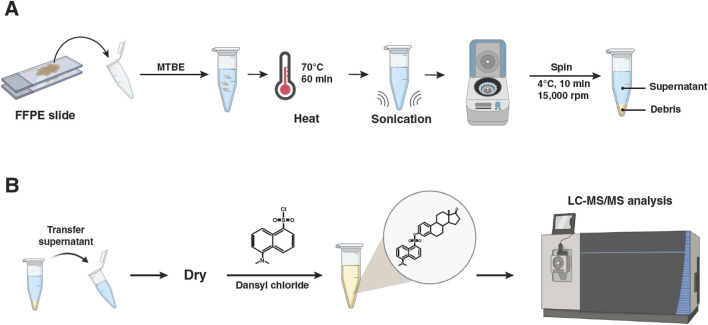
Experimental strategy for the quantitative analysis of estrogen metabolites from formalin-fixed and paraffin-embedded tissues using LC-MS/MS. **(A)** Sample collection and extraction of estrogen metabolites from formalin-fixed and paraffin-embedded tissues. **(B)** Dansylation of extracted estrogen metabolites and LC-MS/MS analysis.

The method used for the targeted analysis of estrogen and its metabolites from FFPE tissue samples is shown in Figure 2. The stromal regions of the MCNs to be collected by scraping were delineated by an expert pathologist, who marked the underside of the slide with a marker. The region of interest on slide-mounted FFPE tissues was scraped from the slide. For normalization purposes, we calculated the area of the scraped normal and the tumor tissue regions using the annotation function of the Zeiss PALM MicroBeam laser capture microdissection microscope system (Zeiss, Thornwood, NY, United States). Extraction and derivatization of estrogen metabolites were conducted following previously described methods, with modifications (Neef et al., 2020; Wojakowska et al., 2015). In brief, two 16-µm-thick tissue sections were scraped from unstained FFPE slides using a razor blade and deposited directly into 1 mL of methyl-tert-butyl ether in a glass vial. Deuterated 17β-estradiol (E2-d2) and estriol (E3-d2) were equally added to all samples as internal standards. The sample was incubated at 70 °C for 10 min to dissolve the paraffin, followed by sonication using Bioruptor Pico (Diagenode, Inc., Denville, NJ, United States) for the extraction of estrogen and its metabolites from tissue. After centrifugation (15,000 rpm for 10 min at 4 °C), the supernatants were transferred to a new vial, and the centrifugation step was repeated to remove residual debris. The clean supernatants were dried in new vials by vacuum centrifugation, followed by dissolution in 20 µL of 0.1 M sodium bicarbonate buffer (pH 9.0) and 20 µL of dansyl chloride solution (1 mg/mL in acetone). After vortexing, the samples were heated at 60 °C for 5 min to form the dansyl derivatives of estradiol, estriol, and other estrogen metabolites (Xu et al., 2007; Nelson et al., 2004).

### Targeted LC-MS/MS analysis

LC-MS/MS analysis was conducted on an Orbitrap Fusion Tribrid ID-X Mass Spectrometer connected to a Vanquish Horizon UHPLC System (Thermo Fisher Scientific, San Jose, CA, United States). The LC separation was performed using a Hypersil GOLD VANQUISH C18 UHPLC column (2.1 × 150 mm, 1.9 µm) using a binary gradient of mobile phase A (0.1% formic acid) and mobile phase B (100% methanol with 0.1% formic acid) at a flow rate of 300 μL/min. Ten microliters of the derivatized samples were injected into the analytical column with 72% mobile phase B. After sample loading, mobile phase B was increased to 85% over 20 min and 100% over 1 min and maintained at 100% for another 5 min. Thereafter, mobile phase B was decreased to 72%, and the analytical column was reconditioned for 4 min. An electrospray voltage of 3.5 kV was fixed in the positive ion mode, and full-scan MS spectra were acquired at a resolution of 60,000 (m/z 200) for a mass range of 250 m/z–800 m/z, and MS/MS spectra were acquired at a resolution of 15,000 (m/z) in scheduled parallel reaction monitoring mode. The molecular ions monitored for the derivatized estrogen and estrogen metabolite species are listed in Supplementary Table S1. The normalized collision energy was set at 42%, automatic gain control was set at 75,000, and ion injection time was 50 ms.

The abundance of individual estrogen metabolites was determined by calculating the peak area ratios relative to the two deuterated standards used as references. Skyline was utilized for peak area calculation (MacLean et al., 2010). The calculated amount of estrogen and its metabolite was normalized to the scraped tissue area to compare the levels of estrogen and its metabolites detected from the same amount of tissue. Significant differences among multiple groups were evaluated using one-way ANOVA, followed by Tukey’s multiple comparison test using MetaboAnalyst 5.0, with *p* < 0.05 considered statistically significant. The T principal component analysis (PCA) plot and heatmap were generated using MetaboAnalyst 5.0 (http://www.metaboanalyst.ca), and abundance plots were visualized using BoxPlotR (http://boxplot.tyerslab.com).

## Results

### Estrogen and its metabolites are detected in pancreatic MCNs

**FIGURE 3:**
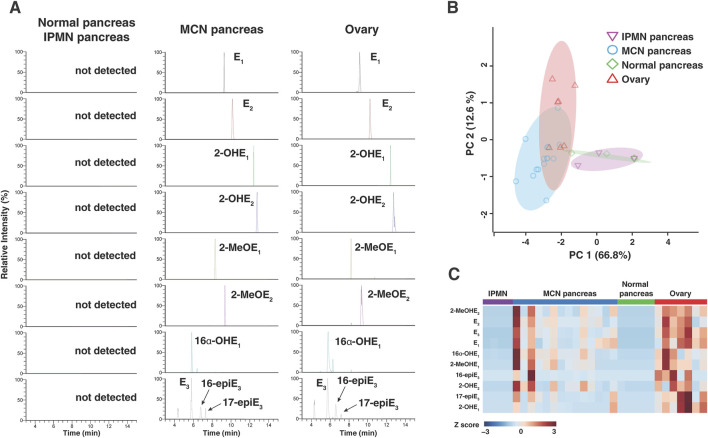
Estrogen and its metabolites in pancreatic tissue from MCNs and control individuals. **(A)** Liquid chromatography–tandem mass spectrometry analysis of estrogen and its metabolites in 16 µm tissue section obtained from normal pancreatic/IPMN pancreatic, MCN pancreatic, and normal ovarian tissues. **(B)** Principal component analysis of estrogen and its metabolite profiles across all the samples, clearly segregating cases with MCN pancreas from normal/IPMN pancreas. **(C)** Heatmap of 10 targeted estrogen metabolites obtained from normal pancreatic, MCN pancreatic, and normal ovarian tissues. 2-OHE_2_, 2-hydroxyestrone; 2-OHE_2_, 2-hydroxyestradiol; 2-MeOE_1_, 2-methoxyestrone; 2-MeOE_2_, 2-methoxyestradiol; 16α-OHE_1_, 16α-hydroxyestrone; E_3_, estriol; 16-epiE_3_, 16-epiesterol; 17-epiE_3_, 17-epiesterol.

Targeted analysis for estrogen metabolites was performed in a 15-min LC-MS/MS assay in parallel reaction monitoring mode. Chromatograms derived from the targeted LC-MS/MS of estrogen and its metabolites observed in normal pancreas, IPMN pancreas, stroma of MCN pancreas, and premenopausal ovarian tissues are shown in Figure 3A. In this chromatogram, 10 endogenous estrogen metabolites were detected from the ovary and pancreatic MCN tissues, but they were not detected in normal pancreatic tissues or pancreatic IPMN tissues, including normal pancreatic tissues from patients with MCNs.

### Mass spectrometry-based analysis shows similar estrogen metabolite profile between MCNs and ovary

The overall differences in estrogen metabolite profiles of normal pancreas (n = 5), IPMN pancreas (n = 4), stroma of MCN pancreas (n = 14), and ovary (n = 7) tissue samples are illustrated using the PCA plot in Figure 3B for all detected estrogen metabolites. Each data point in Figure 3B represents the overall estrogen metabolite profile of individual FFPE tissue samples. The data points for the MCN group are clustered separately from those of the normal pancreas and pancreatic IPMN groups, while the data points for the ovarian group [premenopausal ovaries (n = 3) and ovaries with colorectal cancer metastasis (n = 4)] are clustered nearby. This supports the interpretation that estrogen and its metabolites are present and involved in the biology of pancreatic MCNs in a manner distinct from that of the normal pancreas or other pancreatic cystic neoplasms such as IPMN. Within the MCN group, samples were classified into subgroups (low-grade MCNs, high-grade MCNs, and MCNs with invasive carcinoma) based on the degree of cellular transformation and malignancy risk, and the differences in estrogen metabolite profiles among these subgroups were compared using a PCA plot (Supplementary Figure S1). While the overall estrogen metabolite profile in invasive carcinoma was clustered separately from the other two subgroups (low- and high-grade MCNs), indicating differences, estrogen expression was not found to be significantly higher in carcinoma. In the case of the ovary group, samples were further subdivided into premenopausal normal ovaries and ovaries with CRC metastasis. As shown in the PCA plot, even when comparing only colorectal cancer metastatic to the ovaries to the MCN group, no significant differences in estrogen metabolite profiles were observed (Supplementary Figure S1).

### Quantitative analysis shows that the amount of estrogen and its metabolites in MCNs are significantly different from that in normal pancreas and similar to that in normal ovary

**FIGURE 4:**
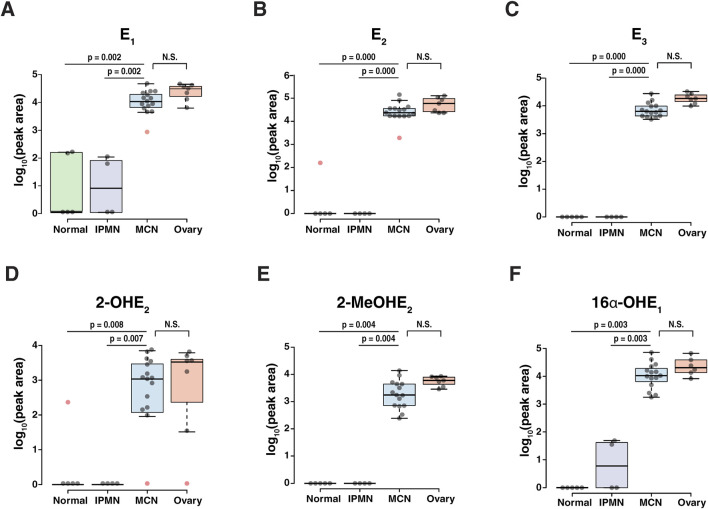
Abundance of estrogen and its metabolites showing significant changes (p-value <0.05) between the normal pancreas/IPMN pancreas and the MCN pancreas. The center lines show the medians; box limits indicate the 25th and 75th percentiles as determined by R software; the whiskers extend to 1.5 times the interquartile range from the 25th and 75th percentiles, and outliers are represented by red dots. **(A)** E1, estrone; **(B)** E2, 17β-estradiol; **(C)** E3, estriol; **(D)** 2-OHE2, 2-hydroxyestradiol; **(E)** 2-MeOE2, 2-methoxyestradiol; **(F)** 16α-OHE1, 16α-hydroxyestrone.

The relative differences in 10 endogenous estrogen metabolites between the four groups were visualized through a heatmap (Figure 3C). The heatmap shows clear differences in the estrogen metabolite profiles between the normal pancreatic or IPMN pancreatic tissues and the stroma of MCN pancreatic or ovarian tissues. Alterations in the level of each estrogen metabolite species within three groups are shown in Figure 4. Of the 10 detected estrogen metabolite species, six species [estrone (E1), estradiol (E2), estriol (E3), 2-hydroxyestradiol (2-OHE_2_), 2-methoxyestradiol (2-MeOE_2_), and 16α-hydroxyestrone (16α-OHE_1_)] (shown in Figure 4) exhibited a significant difference (*p-*value <0.05, as measured by one-way ANOVA, followed by Tukey’s multiple comparison test) between the stroma of MCNs and normal pancreas or IPMN pancreas, whereas the difference between the stroma of MCNs and ovary was not statistically significant. For a more accurate quantitative comparison, the concentration of each detected estrogen metabolite species was calculated using a calibration curve. In addition, the concentration of the estrogen metabolite species was normalized by the scraped area of 16-µm-thick FFPE tissue so that the amount of estrogen metabolite species detected in the same amount of tissue could be compared, as shown in Table 2 and Supplementary Table S2.

**TABLE 2:** Concentration (fmol/1 square millimeters of tissue section) of estrogen and its metabolites in 16 µm tissue sections obtained from pancreatic and ovarian tissues.

Case	Tissue	Diagnosis	Targeted EM species									
			E_1_	E_2_	2-OHE_1_	2-OHE_2_	2-MeOHE_1_	2-MeOHE_2_	16a-OHE_1_	E_3_	16-epiE_3_	17-epiE_3_
1	Pancreas	Low-grade MCN	0.92	3.21	0.73	2.39	0.46	0.79	1.30	1.47	0.30	0.45
2			0.19	0.71	-	0.95	0.10	0.07	0.30	0.41	-	-
3			0.46	1.80	0.22	1.73	0.25	0.50	0.72	0.77	0.39	0.23
4			0.04	0.48	0.22	0.22	0.08	0.12	0.27	0.40	-	0.13
5			0.23	0.86	-	-	0.21	0.11	0.29	0.49	-	-
6			0.27	0.94	0.14	0.93	0.15	0.24	0.40	0.46	0.04	0.11
7			0.12	0.42	0.31	0.31	-	0.07	0.18	0.29	-	-
8			0.10	0.40	0.19	0.46	0.08	0.09	0.16	0.22	-	0.08
9			0.13	0.40	-	0.44	0.08	0.04	0.19	0.26	0.07	0.07
10		High-grade MCN	0.41	0.07	-	-	-	0.04	0.06	0.37	-	0.08
11			0.21	-	-	-	-	0.18	0.04	0.30	-	0.06
12			0.45	0.38	0.29	0.32	-	0.27	0.09	0.47	-	0.19
13		MCN with invasive carcinoma	0.35	0.74	-	0.25	-	0.11	0.05	0.24	-	0.06
14			0.29	0.83	-	1.31	0.13	0.27	0.49	0.71	-	0.13
15		IPMN	-	-	-	-	-	-	-	-	-	-
16			0.01	-	-	-	-	-	0.01	-	-	-
17			-	-	-	-	-	-	-	-	-	-
18			0.01	-	-	-	-	-	0.01	-	-	-
19		Normal	0.02	0.02	-	0.16	-	-	-	-	-	-
20			-	-	-	-	-	-	-	-	-	-
21			0.01	-	-	-	-	-	-	-	-	-
22			-	-	-	-	-	-	-	-	-	-
23			-	-	-	-	-	-	-	-	-	-
24	Ovary	CRC to ovary	0.74	1.89	0.54	0.94	0.11	0.32	-	1.02	-	0.45
25			0.82	2.41	0.61	1.25	0.10	0.43	0.30	1.48	0.21	0.51
26			0.45	-	0.31	0.46	-	0.21	0.18	0.74	-	0.16
27			0.57	0.52	0.41	0.49	0.08	0.29	0.24	0.85	-	0.28
28		Premenopausal	0.12	0.65	0.11	0.92	0.10	0.18	0.42	0.45	0.20	0.07
29			0.71	2.88	0.68	-	0.41	0.50	1.20	1.42	0.31	0.31
30			0.24	1.31	0.18	1.70	0.16	0.41	0.68	0.80	0.34	0.16

Concentration (fmol/square millimeter of tissue section) of estrogen and its metabolites in 16-µm-thick tissue sections obtained from normal pancreatic, IPMN pancreatic, MCN of pancreatic, and premenopausal ovarian tissues. “-,” not detected.

### The levels of estrogen and its metabolites in MCN tumors are independent of tumor size and patient age

**FIGURE 5:**
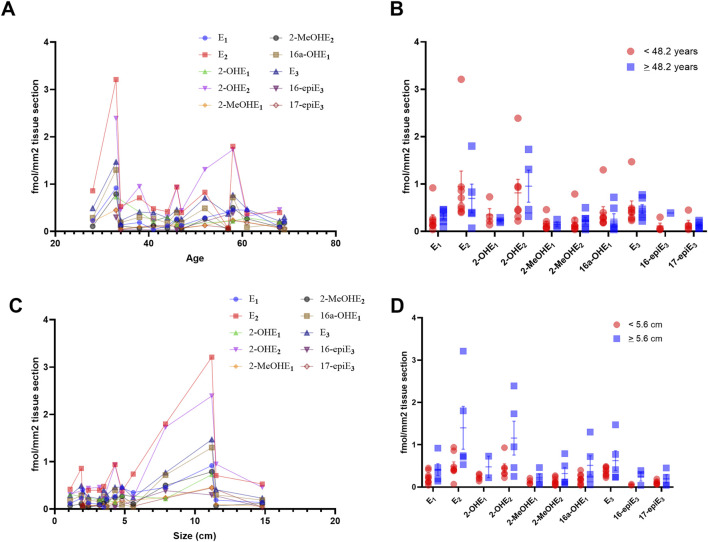
Levels of estrogen and its metabolites in MCN tumors are independent of tumor size and patient age. **(A)** Spearman correlation coefficient, comparing the concentration of estrogen and its metabolites per mm^2^ and the age of the patient, demonstrating no significant correlation with age. **(B)** No significant difference was observed between patients above or below the mean age of our cohort. **(C)** Spearman correlation coefficient, comparing the concentration of estrogen and its metabolites per mm^2^ and the size of the resected tumor. Size was significantly positively correlated with levels of 2-MeOHE_2_ (r = 0.54; *p* = 0.049). No other significant correlation was identified. **(D)** No significant difference was observed between tumors larger or smaller than the mean tumor size of our cohort. Significance for B and D calculated using multiple t-tests, adjusted for multiple comparisons using two-stage step-up (Benjamini, Krieger, and Yekutieli) with a false discovery rate (FDR) of 0.05.

Within our cohort of patients with MCN tumors, the mean age was 48.3 (±13) years, and the mean tumor size was 5.56 (±4.2) cm. No correlation was observed between the age of our patients and the size of the resected tumor (*p* = 0.64). We observed no significant correlation between age and the levels of estrogen metabolites detected in the tissues (Figure 5A), and no differences were found between patients above or below the mean age (Figure 5B). Compared to the size of the tumor and the levels of estrogen metabolites detected in the tissues, we identified that size was significantly positively correlated with levels of 2-MeOHE_2_ (r = 0.54; *p* = 0.049), a naturally occurring metabolite of estrogen. No other significant correlation was identified (Figure 5C), and no differences were observed between patients with tumor size above or below the mean tumor size (Figure 5D). These highly interesting findings suggest that the levels of estrogen and its metabolites per square millimeter of area remain independent of patient age; however, some metabolites may demonstrate a correlation with tumor size.

## Discussion

Mucinous cystic neoplasms of the pancreas are characterized by glandular columnar epithelium with adjacent ovarian-type stroma. In the current study, we describe the detection of estrogen and its metabolites in tissue from mucinous cystic neoplasms through a novel mass spectrometry technique. These findings represent an important investigation into the functional nature of the ovarian-type stroma in pancreatic mucinous neoplasms and demonstrate the homology between MCNs and normal ovarian-type stroma in their capability to produce estrogen.

Our current study shows that relative to histologically normal pancreas and other mucinous neoplasms of the pancreas (IPMN), the stroma in MCNs expresses significantly greater levels of estrone (E_1_), estradiol (E_2_), estriol (E_3_), 2-hydroxyestradiol (2-OHE_2_), 2-methoxyestrone (2-MeOE_1_), 2-methoxyestradiol (2-MeOE_2_), and 16α-hydroxyestrone (16α-OHE_1_). Furthermore, these metabolites were present at levels similar to those observed in the stroma in the normal ovary (Figure 4; Table 2) and the levels of estrogen in colorectal cancer metastatic to the ovary (Supplementary Figure S1B). Combined with previously published molecular signatures, which demonstrate that MCN tumors show significantly increased expression of key steroidogenic enzymes such as aromatase (CYP19A1), 17α-hydroxylase/17,20 lyase/17,20 desmolase (CYP17A1), 17β-hydroxysteroid dehydrogenase 1 (HSD17B1), steroidogenic acute regulatory protein (Star), and cholesterol side chain cleavage enzyme or cholesterol desmolase (CYP11A1) (Van Treeck et al., 2020), this provides convincing evidence that the stroma of MCNs produces estrogen locally, similar to what is observed in the normal ovary.

In view of the constancy of ovarian-type stroma in this tumor type (as required by its definition), these findings raise the question of whether local estrogen production is important to the pathogenesis of MCNs. The lack of detection of these markers in the normal pancreas from premenopausal women excludes the possibility of false detection. Multiple studies have also demonstrated the expression of estrogen receptors in this stroma, which may represent a therapeutic target if estrogen is mechanistically linked to tumor progression. (Van Treeck et al., 2020; Ishida et al., 2016; Pflüger et al., 2025; Pea et al., 2025).

Given that MCN is an uncommon precursor of invasive adenocarcinomas, these data also raise the possibility that estrogen plays a role in the pathogenesis of at least some subsets of invasive malignancies. Notably, simple biliary cysts and simple mucinous cysts of the pancreas are not believed to have any malignant potential and resemble MCNs with the key exception of the lack of an ovarian-type stroma. This observation heightens interest in the possibility that ovarian-type stroma, and now estrogen production, may be relevant to the risk of malignant progression in this histological context, either as a result of direct hormonal stimulation or through the creation of a privileged pro-tumoral environment similar to that observed in the ovary. Elevated estrogen levels have been demonstrated to contribute to the proliferation of tumors in the ovary and breast (Spillman et al., 2010; Kim and Munster, 2025). Elevated serum estrogen has previously been demonstrated to stimulate the proliferation of human cholangiocarcinoma, which similarly expresses estrogen receptors in tumoral tissue (Mancino et al., 2009; Hunsawong et al., 2012; Petrick et al., 2020).

Previous studies on a large patient cohort of 163 patients established that benign MCNs were significantly smaller than malignant tumors and that patients with invasive carcinoma were significantly older than those with non-invasive carcinoma (Crippa et al., 2008). Our findings highlight that no significant correlation exists between patient age and estrogen or its metabolites. We identified that 2-me-OHE_2_ was significantly correlated with tumor size; however, no other significant correlations were identified. Taken together, our data suggest that the overall hormonal functionality in the ovarian-type stroma of MCNs remains relatively stable over time and tumor size.

Multiple studies in colorectal adenocarcinomas have now established the ovary as a sanctuary for metastases, with tumor deposits in the ovary responding poorly to systemic chemotherapy and resulting in worse overall survival outcomes. The effects of estrogen on the tumor microenvironment in these metastases could partly be responsible for the observed differential response to treatment. Estrogen has also been demonstrated to modulate immune response, favoring an immunosuppressive M2 polarization in macrophages and an immunosuppressive regulatory T-cell phenotype in CD4 cells. Studies on epithelial ovarian cancers have demonstrated decreased tumor-infiltrating lymphocyte (TIL) activity due to the effects of regulatory T-cells (Tregs), myeloid-derived suppressor cells (MDSCs), and tumor-associated macrophages (TAMs). Our demonstration of endogenous estrogen in the MCN stroma may explain prior observations of increased transcripts corresponding to regulatory T-cells in MCNs (Tai et al., 2008). The similarity observed between MCN stroma and the normal ovary raises the possibility that estrogen production is linked to the immune microenvironment, which is conducive to tumor development.

Ovarian stroma has also been described as having significantly increased expression of markers of sex-steroid differentiation, with steroidogenesis capable of converting androgens into estrogens in ovarian epithelial neoplasms and being correlated with the progression of epithelial ovarian tumors. In ovarian tumors, it has been postulated that the steroidogenic capability of the stroma, coupled with the expression of the corresponding receptors in the epithelial component of the tumor, may contribute to tumorigenesis (Tokunaga et al., 2007; Kato et al., 2013; Blanco et al., 2017). It has previously been shown that the stroma of mucinous cystic ovarian neoplasms similarly expresses strong reactivity for estrogen receptor (ER) and progesterone receptor (PR), possessing occasional luteinized stromal cells and similar overexpression of steroidogenic markers and enzymes in ovarian mucinous adenocarcinomas; it has been suggested that SF-1 overexpression may activate aromatase and promote estrogen biosynthesis. A mechanistic investigation of this phenomenon is beyond the scope of this study and remains to be fully elucidated; however, in combination with the published literature, our proteomic findings indicate that a similar pathway of estrogen production may occur in MCNs of the pancreas (Hattori et al., 2015).

Our current study is descriptive in nature and aims to characterize the novel biology of the ovarian-type stroma in MCN tumors; however, it is limited by a small sample size and by its scope, which focuses solely on mucinous cystic neoplasms of the pancreas. The descriptive nature of the study and the small sample size precludes multi-variate analysis, which would be necessary to definitively conclude whether the levels of estrogen and its metabolites can serve as independent variables, alongside tumor size and patient age, in predicting tumor progression from low-grade dysplasia to adenocarcinoma and prevent the generalizability of this data. Furthermore, our study does not investigate the systemic effects of tumoral estrogen production, which may have implications in other organ systems.

Although this study represents a significant step forward in our understanding of the functional biology of the ovarian-type stroma in MCN tumors, future studies are required to determine the exact mechanistic role of the endogenous and exogenous estrogen in the pathogenesis of these tumors and whether the estrogen produced by these tumors may result in systemic effects on the patient. This study is also limited by the lack of cases in male individuals, given their rarity.

Overall, this study demonstrates, through a novel mass spectrometric technique, the first detection of local estrogen production in the ovarian-type stroma of mucinous cystic neoplasms of the pancreas, and it has implications in our understanding of the importance of estrogen in the pathogenesis of these tumors and the estrogen biosynthetic pathway as a potential therapeutic target.

## Data Availability

The original contributions presented in the study are included in the article/supplementary material, further inquiries can be directed to the corresponding author.

## Supplementary material

SUPPLEMENTARY FIGURE S1 Principle component analysis of estrogen and its metabolite profiles for comparison **(A)** between subgroups of MCN pancreas (low-grade MCN vs. high-grade MCN vs. MCN with invasive carcinoma) and **(B)** between MCN pancreas and ovaries with colorectal cancer metastasis (CRC to ovary).

SUPPLEMENTARY TABLE S1 Precursor and quantifier ions used for each targeted estrogen metabolite (dansylated) species for PRM quantification by UHPLC-MS/MS.
