# Supplementary material for: Mucinous cystic neoplasms of the pancreas demonstrate in situ production of estrogen
Source: Front Cell Dev Biol. 2025 Nov 17;13:1684564. doi: 10.3389/fcell.2025.1684564 (PMC12665673; doi:10.3389/fcell.2025.1684564)
Supplement: Supplementary file 1 [file DataSheet1.pdf]

Figure S1.

A

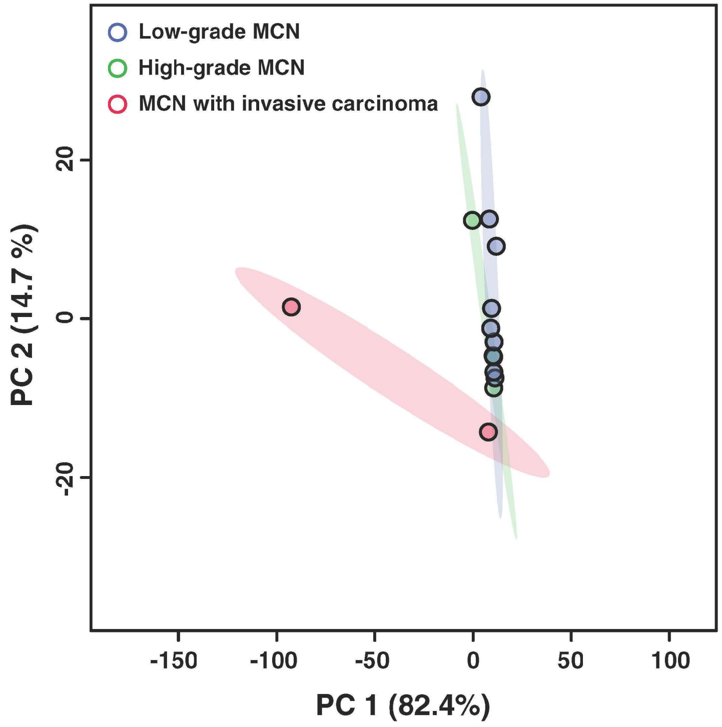
Low-grade MCN
High-grade MCN
MCN with invasive carcinoma
20
0
-20
PC 2 (14.7 %)
-150
-100
-50
0
50
100
PC 1 (82.4%)

B

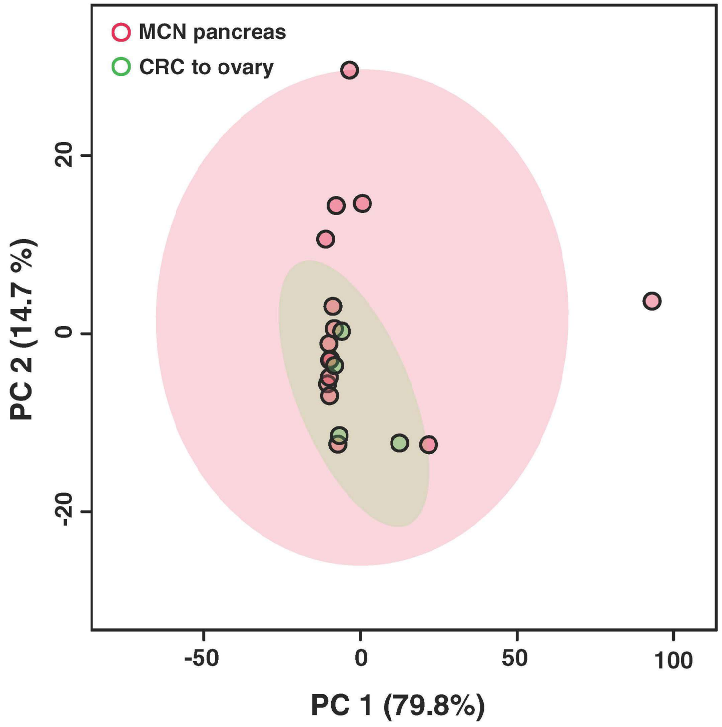
MCN pancreas
CRC to ovary
20
0
-20
PC 2 (14.7 %)
-50
0
50
100
PC 1 (79.8%)

TABLE S1

Estrogen metabolites	precursor ion (*m/z*)	quantifier ion (*m/z*)
Estrone	504.2203	171.1044
17β-Estradiol	506.2359	171.1044
Estriol	522.2303	171.1044
16-Epiestriol	522.2303	171.1044
17-Epiestriol	522.2303	171.1044
16α-Hydroxyestrone	520.2146	171.1044
2-Methoxyestrone	534.2303	171.1044
4-Methoxyestrone	534.2303	171.1044
2-Methoxyestradiol	536.2459	171.1044
4-Methoxyestradiol	536.2459	171.1044
2-Hydroxyestrone	753.2651	170.0966, 519.2080
4-Hydroxyestrone	753.2651	170.0966, 519.2080
2-Hydroxyestradiol	755.2808	170.0966, 521.2229
17β-estradiol-d_2	508.2480	171.1044
Estriol-d_2	524.2429	171.1044

Table S2. Everage concentration (fmol/1 square millimeters of tissue section) of estrogen and estrogen metabolites in 16 µm tissue section obtained from pancreas and ovary tissues

Tissue	Diagnosis	Number of samples	Targeted EM species									
			E_1	E_2	2-OHE_1	2-OHE_2	2-$MeOHE_1$	2-$MeOHE_2$	16a-OHE_1	E_3	16-epiE_3	17-epiE_3
pancreas	Low-grade MCN	9	0.27	1.02	0.20	0.83	0.16	0.23	0.42	0.53	0.09	0.12
	High-grade MCN	3	0.36	0.15	0.10	0.11	-	0.16	0.06	0.38	-	0.11
	MCN with invasive carcinoma	2	0.32	0.79	-	0.78	0.07	0.19	0.27	0.48	-	0.10
	IPMN	4	0.01	-	-	-	-	-	0.01	-	-	-
	Normal	5	0.01	-	-	0.03	-	-	-	-	-	-
ovary	CRC to ovary	4	0.65	1.21	0.47	0.79	0.07	0.31	0.18	1.02	0.05	0.35
	pre-menopausal	3	0.36	1.61	0.32	0.87	0.22	0.36	0.77	0.89	0.28	0.18
